# Detection of *Schistosoma mansoni* Eggs in Feces through their Interaction with Paramagnetic Beads in a Magnetic Field

**DOI:** 10.1371/journal.pntd.0000073

**Published:** 2007-11-14

**Authors:** Candida Fagundes Teixeira, Erli Neuhauss, Renata Ben, Juliano Romanzini, Carlos Graeff-Teixeira

**Affiliations:** Laboratórios de Parasitologia Molecular e de Biologia Parasitária, Instituto de Pesquisas Biomédicas e Faculdade de Biociências, PUCRS, Porto Alegre, Brazil; George Washington University, United States of America

## Abstract

**Background:**

Diagnosis of intestinal schistosomiasis in low endemic areas is a problem because often control measures have reduced egg burdens in feces to below the detection limits of classical coproparasitological methods. Evaluation of molecular methods is hindered by the absence of an established standard with maximum sensitivity and specificity. One strategy to optimize method performance, where eggs are rare events, is to examine large amounts of feces. A novel diagnostic method for isolation of *Schistosoma mansoni* eggs in feces, and an initial evaluation of its performance is reported here.

**Methodology/Principal Findings:**

Known amounts of *S. mansoni* eggs were seeded into 30 g of normal human feces and subjected to a sequence of spontaneous sedimentation, sieving, Ritchie method, incubation and isolation through interaction with paramagnetic beads. Preliminary tests demonstrated the efficacy of lectins as ligands, but they also indicated that the paramagnetic beads alone were sufficient to isolate the eggs under a magnetic field through an unknown mechanism. Eggs were identified by microscopic inspection, with a sensitivity of 100% at 1.3 eggs per gram of feces (epg). Sensitivity gradually decreased to 25% at a concentration of 0.1 epg. In a preliminary application of the new method to the investigation of a recently established focus in southern Brazil, approximately 3 times more eggs were detected than with the thick-smear Kato-Katz method.

**Conclusions/Significance:**

The novel *S. mansoni* detection method may significantly improve diagnosis of infections with low burdens in areas of recent introduction of the parasite, areas under successful control of transmission, or in infected travelers. It may also improve the evaluation of new treatments and vaccines.

## Author Summary

*Schistosomiasis mansoni* is a parasitic infection that affects approximately 200 million people, mainly in the tropics. The worms live inside the veins of intestines and liver and produce eggs that are eliminated within feces. If the eggs reach water, a ciliated larva is released and enters snails to develop into a larva infective to man and other vertebrates. Most infections evolve without overt disease, but severe intestinal, hepatic, pulmonary and cerebro-medulary dysfunctions may occur after many years. Definitive diagnosis is made through the identification of eggs in stool. Classical diagnostic methods fail to detect infection when the number of eggs is low (e.g., in areas where control measures have decreased the intensity of infection or in the case of light infections in travelers who have had only brief exposure). A new and very sensitive method is reported here, in which eggs are isolated from large amounts of feces through their interaction with magnetic beads. After incubation with the fecal sediment, eggs co-migrate with the beads towards a magnet attached to the test tube. This improvement in diagnostic methodology will strengthen efforts to control schistosomiasis.

## Introduction

In areas with recent introduction of *Schistosoma mansoni* transmission or areas where control efforts have reduced parasitic burden, the classical parasitological methods for finding eggs in stools do not demonstrate sufficient sensitivity [1],[2],[3]. Travelers may have light *S. mansoni* infections of difficult definitive diagnosis [4]. Molecular diagnostic tools are an alternative detection method, but more extensive validation in areas of low endemicity is still lacking. Very sensitive egg detection systems are desirable as methods for definitive diagnosis and are the standard for evaluation of other indirect diagnostic methods, such as serological tests. Since *S. mansoni* eggs are large and have a peculiar shape and a lateral spine, they are easily recognized visually, using a microscope, leading to a definitive diagnosis with few possibilities of false-negative results. Increased amounts and/or numbers of fecal samples, the use of differential fecal concentration methods or even application of mathematical modeling have been tested in order to improve sensitivity of parasitological diagnosis, but none of these approaches have been convincing enough to warrant extensive field trials [5],[6].

Paramagnetic beads coupled to a variety of ligands are available for different applications such as purification of whole cells, organelles, nucleic acids, proteins and other molecules [7],[8], and may be used for *S. mansoni* antigen detection [9]. Magnetic separation has also been applied for parasitological diagnosis, for example, in detection of cryptosporidiosis and giardiasis [10]. Here we report the preliminary evaluation of a highly sensitive method for isolation and detection of *S. mansoni* eggs from large amounts of feces which is based on their interaction with paramagnetic beads in a magnetic field.

## Materials and Methods

Laboratory samples of *Schistosoma mansoni* were maintained by passages through Swiss albino mice and *Biomphalaria glabrata*. Both parasite and molluscs came from a local “Esteio” strain (Rio Grande do Sul, Brazil). Eggs were recovered by artificial digestion of livers, from experimentally infected rodents, in 4% KOH for 2 h at 46°C.

### Experiments with lectins as ligands

Stock solutions (1 mg/mL) of five biotinilated lectins were prepared with a pH 7.6 “lectin buffer” (6.057 g TRIS, 8.7 g NaCl, 0.203 g MgCl_2_, 0.111 g CaCl_2_ and 0.02% sodium azide, per Litre) [11]. The five lectins (SIGMA, USA), reported in the literature as ligands to the surface of *S. mansoni* eggs [12],[13],[14], were: *Triticum vulgaris* (L5142), Concavalin A (C2272), *Ulex europeaus* (L8262), *Arachis hypogea* (L6135), and *Lycopersicum esculentum* (L0651). Microtubes containing 100 eggs in 100 µL PBS plus 50 µL of lectins at several concentrations (5 µg/mL, 10 µg/mL and 20 µg/mL) were incubated at room temperature for 1 h. After a washing step with PBS, the volume was adjusted to 1.5 mL and paramagnetic beads covered with streptavidin (Bangs Labs, USA) were added to a final concentration of 1.4% (v/v). Incubation was performed in an orbital shaker, at room temperature, for 1 h. Eggs and beads, without lectins, were incubated as a negative control. The microtubes containing the preparations were connected to a magnet (Dynal, Oslo, Norway) for 3 min, supernatants were removed, and the sediments retained at the wall were collected and examined under a microscope for counting of *S. mansoni* eggs. Several combinations of lectins were also tested under the same conditions and at a final individual concentration of 40 µg/mL.

### Experiment with different beads

Paramagnetic beads coupled with 1) anti-rabbit-IgG (Dynal, Oslo, Norway); 2) a monoclonal antibody anti-Cryptosporidium (Dynal, Oslo, Norway); 3) streptavidin (Bangs Lab, USA); 4) streptavidin followed by biotinylated lectin (SIGMA, USA), were incubated with eggs in distilled water, under the conditions described above. A preparation with eggs and without beads (negative control) and another with eggs and a latex (non-magnetic) bead coupled to Protein A (Dynal, Oslo, Norway) were also tested.

### Seeding experiment in feces with and without lectins

Based on the results from the previous experiment, 10 *S. mansoni* eggs were seeded in 30 g of normal human feces. The fecal sample was suspended and stirred in 250 mL of water, filtered through 8 layers of surgical gauze into a conical cup, left for 1 h at RT, re-suspended in distilled water and the spontaneous sedimentation repeated until a clear supernatant was obtained. Sediment was sieved through 100 (S1), 200 (S2) and 325 (S3) meshes per square inch metal sieves. The fraction retained at S3 was submitted to the method of Ritchie [15] and the procedure was repeated usually twice or as necessary to get a clear supernatant without a ring of debris. The final sediment was incubated either with or without 20 µg/mL of biotinylated *Triticum vulgaris* as previously described in the lectin experiments. Paramagnetic beads were coupled with streptavidin (Bangs Lab, USA).

### Seeding experiment to evaluate sensitivity

Eggs were added at different concentrations (60, 40, 30, 20, 10, 7 and 3) per 30 g of normal human feces, corresponding respectively to 2.0, 1.3, 1.0, 0.6, 0.3, 0.2, 0.1 eggs per gram of feces (epg). The fecal samples were processed as described above, except that lectins were not employed and uncoated paramagnetic beads (BioMag, BM547/7065, Bangs Lab, USA) were used to isolate the eggs.

The novel method was employed for the first time in the investigation of two infected individuals from a potential new focus of transmission in Porto Alegre, the capital of Brazil's southernmost State. For each sample (whole evacuation) 2 thick fecal smears or Kato-Katz method [16] were prepared and 30 g of feces were processed with the new method. The method and kit, including the reagents, magnet and sieves, have been named Helmintex (patent pending).

### Ethical oversight

The study protocol was approved by the ethics committee of the Public Health Central Laboratory of Rio Grande do Sul (LACEN/RS). The normal feces used in the seeding experiments were donated by three of the authors (C G-T, CFT, and JR). Informed consent was obtained from the two infected individuals who donated feces for the study.

## Results

Table 1 shows the percentage of eggs found in the supernatant (SN) and in the pellet (SD) formed at the wall of the microtube in contact with the magnet. Both the negative control and the several preparations with lectins contained most of the eggs in the pellet. The results indicated that both negative control and several combinations of lectins were effective at promoting isolation of the eggs (Table 2). The data in Table 3 were used to compare treatments with different beads, and confirmed that lectins were not essential and that paramagnetic beads were necessary for isolation of the eggs, since latex beads and eggs alone did not migrate to the magnet. The results of the seeding experiment in feces with and without lectins (data not shown), besides confirming that lectins were not essential, demonstrated that isolation of eggs occurred in the presence of fecal sediment. Based on these initial results we decided to use uncoupled beads for future testing.

**Table 1:** Percentage of *S. mansoni* eggs recovered in supernatant (SN) and pellet (SD) after seeding experiments with different concentrations of biotinilated lectins and paramagnetic beads covered with streptavidin.

Lectins	SN or SD	0 µg/mL	5 µg/mL	10 µg/mL	20 µg/mL
***Triticum vulgaris***	SN (%)	44	17	10	0
	**SD** (%)	**56**	**83**	**90**	**100**
***Concavalim A***	SN (%)	1	3	3	15
	**SD** (%)	**99**	**97**	**97**	**85**
***Ulex europeaus***	SN (%)	23	4	18	7
	**SD** (%)	**77**	**96**	**82**	**93**
***Arachis hypogaea***	SN (%)	0	5	4	7
	**SD** (%)	**100**	**95**	**96**	**93**
***Lycopersicum***	SN (%)	5	3	14	3
***esculentum***	**SD** (%)	**95**	**97**	**86**	**97**

**Table 2:** Percentage of *S. mansoni* eggs recovered in supernatant (SN) and pellet (SD) after seeding experiments with different combinations of biotinilated lectins and interaction with paramagnetic beads covered with streptavidin.

Lectins	SN	SD
Ca+Ue	3	**97**
Ca+Ah	5	**95**
Ca+Tv	23	**77**
Negative control	1	**99**
Eu+Ah	6	**94**
Ca+Le	6	**94**
Eu+Tv	7	**93**
Negative control	2	**98**
Eu+Lê	4	**96**
Ah+Tv	2	**98**
Ah+Lê	2	**98**
Tv+Lê	1	**99**
Negative control	27	**73**

For every of three experiments, there was a negative control: eggs and beads without lectins.

*Triticum vulgaris* (Tv); *Concavalim A* (Ca); *Ulex europeaus* (Ue); *Arachis hypogaea* (Ah); *Lycopersicum esculentum* (Le)

**Table 3:** Percentage of *S. mansoni* eggs recovered in supernatant (SN) and pellet (SD) after three repetitions of seeding experiments: paramagnetic microspheres covered with different ligands, latex beads covered with Protein A and egg suspension as negative control.

Repeat	SN or SD	Negative control	Paramagnetic beads covered with			Latex beads covered with Protein A	Positive control
			Antibody anti-rabbit IgG	Streptavidin	Antibody anti-Cryptosporidium		
1st	SN	99	36	3	34	98	1
	**SD**	**1**	**64**	**97**	**66**	**2**	**99**
2nd	SN	100	24	3	55	94	0
	**SD**	**0**	**76**	**97**	**45**	**6**	**100**
3rd	SN	91	39	0	57	99	6
	**SD**	**9**	**61**	**100**	**43**	**1**	**94**

A preparation resulting from the incubation of eggs with biotinilated lectins and interaction with magnetic beads covered with streptavidin was also included as a positive control.

The recovery of seeded eggs in fecal samples is shown in Table 4. Sensitivity was 100% with egg burdens of 2 and 1.3 epg and gradually decreased to 20% as the egg burden was reduced to 0.1 epg. The investigation of the new focus in Porto Alegre revealed one infection (Patient A) out of 6 individuals of the family and the confirmation of infection in the index case (Patient B), with total numbers of eggs per sample as shown in Table 5.

**Table 4:** Eggs of *S. mansoni* recovered after seeding experiments where different numbers of eggs were mixed with 30g of normal human feces.

Eggs per gram (Total eggs per 30 g)	3 (0.1)	7 (0.24)	10 (0.34)	20 (0.67)	30 (1.0)	40 (1.34)	60 (2)
**Repetitions**							
1	0	1	1	3	3	4	5
2	0	1	0	1	3	2	3
3	0	2	0	0	4	7	6
4	1	0	0	0	1	1	2
5	0	0	0	0	0	3	2
6	0	3	0	2	5	10	3
7	1	0	2	0	7	5	nd (*)
8	0	0	2	3	3	6	nd
9	0	0	3	1	0	5	nd
10	0	3	2	1	4	5	nd
**Total eggs**	2	10	10	11	30	48	21
**Recovery (%)**	6.7	14.3	10.0	5.5	10.0	12.0	5,9
**Sensitivity**	**20%**	**50%**	**50%**	**60%**	**80%**	**100%**	**100%**

(*) nd: not done

**Table 5:** Identification of *S. mansoni* eggs by the methods of Kato-Katz and Helmintex in samples from two infected individuals from a new focus of intestinal schistosomiasis in Porto Alegre, southern Brazil.

Patients	Kato-Katz	Helmintex
**Patient B (index case)**		
Sample 1	2 slides-negative	3 slides–1 egg
Sample 2	2 slides–1 egg	8 slides–61 eggs
Sample 3	2 slides–2 eggs	10 slides–29 eggs
**Totals**	**6 slides–3 eggs**	**21 slides–91 eggs**
**Patient A**		
Sample 1	2 slides–negative	10 slides–negative
Sample 2	2 slides–negative	10 slides–1 eggs
Sample 3	2 slides–negative	10 slides–24 eggs
**Totals**	**6 slides–0 eggs**	**30 slides–25 eggs**

## Discussion

The Kato-Katz (KK) method is the cornerstone for parasitological diagnosis of *S. mansoni* infection. The KK method has the advantage of being a simple and inexpensive procedure, which has justified its widespread use in the classical endemic areas of intestinal schistosomiasis [17]. Several efforts have been made to develop more sensitive diagnostic tools, such as immunoassays for detection of *S. mansoni* antibodies [1] or antigens [18], PCR [19] methods, and examination of large amounts of feces with isolation of eggs in a Percoll gradient [5]. Molecular methods have not been properly evaluated for sensitivity since current parasitological methods themselves lack sensitivity and few of them have undergone extensive field evaluations [17]. Assays for detection of antigens may not have the expected high sensitivity and specificity when employed for diagnosis in low endemicity areas or in light infections of travelers [18],[20].

In the southernmost transmission focus of schistosomiasis in Brazil, most of the infected individuals examined had less than 1 epg and were usually diagnosed only after examination of several samples with increasing amounts of feces [2]. Efforts to develop a much more sensitive detection method originated with the idea of collecting and examining the entire evacuation of individuals who are of high epidemiological risk but who have consistently tested negative by coproparasitological examinations.

Attempts were made to isolate eggs from large final fecal sediments using sucrose density columns and paramagnetic beads covered with anti-*S. mansoni*-egg-surface antibodies raised in rabbits without success (data not shown). Using the Helmintex method described here, efficient isolation was achieved when lectins were used as ligands to the paramagnetic beads. Isolation of the eggs was also unexpectedly achieved when only beads and eggs were incubated (negative control), suggesting that ligands were unnecessary for isolation of the eggs. Although a detailed explanation of the mechanism of isolation is lacking, it clearly depends on the magnetic field and the presence of paramagnetic beads since eggs alone did not migrate to the magnet. It is possible that the eggs were carried along with the beads as they aligned themselves with the force of the magnetic field and moved towards the magnet. It is not a highly specific interaction since a substantial amount of fecal debris also migrated towards the magnet. However, the reduction in the volume of final sediment and the concentration of eggs in this sediment appeared to be the basis for success of this very sensitive method.

The amount of feces (30 g) used for this test was arbitrarily chosen. It is anticipated that ongoing field tests of the method will demonstrate whether amounts larger than 30 g should be examined. This novel method is far more sensitive then other existing coproparasitological tests. Nevertheless it is expected that additional modifications could be made that may further improve the performance of the Helmintex method. Although Helmintex is a relatively expensive (US$ 0.80 per sample) and laborious method, it is more sensitive than KK. This method was able to detect 1.3 epg with 100% sensitivity while studies of KK indicate that its sensitivity is reduced to 60% at egg burdens lower than 100 epg [3]. Data presented in Table 5 indicated that approximately 30 times more eggs were recovered using Helmintex, than using KK, resulting in recoveries of 91 and 25 for Helmintex compared to 3 and 0 for KK, in patients B and A, respectively. The best performance of the egg hatching method was reported in the literature to have a sensitivity of 100% with 12 epg and 80% with 1 epg [21].

This novel detection method is not meant to replace other classical methods (e.g., thick smear KK) for *S. mansoni* screening. However, in low endemicity areas it may be part of a series of screening steps including epidemiological surveys with quantification methods of risk behavior, KK and serology. For case-control studies Helmintex will be useful as criterion of uninfected groups and, in travelers, it will improve the ability to make a definitive diagnosis in an ever increasing amount of people returning home after brief contact with transmission foci abroad resulting in a very light infection [3]. It may also serve as an extremely valuable tool to be used as the standard for evaluation of other diagnostic tests and vaccines.
